# Supplementary material for: Seroprevalence of brucellosis and molecular characterization of Brucella spp. from slaughtered cattle in Rwanda
Source: PLoS One. 2022 Nov 22;17(11):e0261595. doi: 10.1371/journal.pone.0261595 (PMC9681097; doi:10.1371/journal.pone.0261595)
Supplement: Attachment — Submitted filename: Response to reviewers.docx [file pone.0261595.s002.docx]

**Response to reviewers**

**July 30^th^, 2022**

**Editor, Reviewer 1 and Reviewer 2**

**PLOS One**

**Re: revision of the manuscript [PONE-D-21-38427]-[EMID: 82b0bed3761197ba]**

Dear Editor and reviewers,

The authors would like to acknowledge your valuable inputs. We thank you for reviewing our manuscript entitled “**Seroprevalence of brucellosis and molecular characterization of *Brucella* spp. from slaughtered cattle in Rwanda**”. Your comments were very clear and useful to improve the quality of this manuscript. We have revised the manuscript considering all issues mentioned in your comments. Outlined below are the point-by-point responses.

**Editor**

**Comment:** line 26-27: parallel interpretation means positive in at least one test. So, what the authors mentioned is not parallel but the result of serial interpretation i.e, positive in both tests.

**Response:** the comment was addressed. Parallel replaced by series

**Comment:** line 56: It was not clear what the authors wanted to mean by “As brucellosis is a herd disease”

**Response:** the sentence was rephrased as follows: “to detect brucellosis at herd level, the ……”

**Comment:** line 105: 0.5% should be 5%; what was the basis to consider a 5% expected prevalence? "The expected prevalence was estimated at 0.5%." Here the word "estimated" is not correct.

**Response:** the comment was addressed. 0.5% replaced by 5% and the sentence was rephrased as follows: “the expected prevalence was 5% based on previous studies (Ntivuguruzwa et al., 2020)”.

**Comment:** line 200: Brucellosis seroprevalence among slaughter cattle in Rwanda; this subsection can be replaced by “Descriptive statistics” and lines 201 to 205 could be placed under this subsection.

Then the remaining lines can be placed under another sub-section like Brucellosis seroprevalence.

**Response:** the comment was addressed. A subsection of descriptive statistics was added

**Comment:** please insert another table [preferably Table 2] summarizing all test results

**Response:** a separate table 2 was added as follows:

Table 2. Serological, bacterial culture and PCR results of samples collected from slaughtered cattle in Rwanda

| **Tested** | **RBT** | **i-ELISA** | **RBT&i-ELISA** | **Culture** | **ITS PCR** | **AMOS PCR** | | | **Bruce-ladder PCR** | |
| --- | --- | --- | --- | --- | --- | --- | --- | --- | --- | --- |
| **N** | **n^+^ (%)** | **n^+^ (%)** | **n^+^ (%)** | **n^+^(%)** | **n^+^(%)** | **B. abortus** | **B. melitensis** | **B. abortus&B. melitensis** | **B. abortus** | **B. melitensis** |
| 300 | 62 (20.7) | 8 (2.7) | 8 (2.7) | 87 (29.0) | 17 (5.7) | 4 | 3 | 4 | 5 | 6 |

**Comment:** Please define brucellosis case status [preferably if culture or PCR positive] and only show the distribution of the brucellosis according to the different variables presented in Table 2 deleting the distribution of brucellosis based on RBT and i-ELISA [now it will become Table 3].

**Response:** table was readjusted as follows:

| **Variables** | **Categories** | **Tested** | **ITS PCR assay on culture isolates** | | |
| --- | --- | --- | --- | --- | --- |
|  |  |  | **n^+^ (%)** | **Odds Ratio** | ***p-*value** |
| Abattoirs | High throughput | 280 | 16 (5.71) | 0.02-6.22 | 1 |
|  | Low throughput | 20 | 1 (5.00) |  |  |
| Provinces | Eastern | 70 | 2 (2.86) | Undetermined | 0.43 |
|  | Kigali city | 30 | 3 (10.00) |  |  |
|  | Northern | 50 | 4 (8.00) |  |  |
|  | Southern | 80 | 3 (3.75) |  |  |
|  | Western | 70 | 5 (7.14) |  |  |
| Age | Adults | 269 | 17 (6.32) | 0.00-2.09 | 0.23 |
|  | Young | 31 | 0 (0.00) |  |  |
| Sex | Females | 287 | 17 (5.92) | 0.00-5.75 | 1 |
|  | Males | 13 | 0 (0.00) |  |  |
| Breed | Ankole | 83 | 1 (1.20) | Undetermined | 0.02 |
|  | Cross | 201 | 13 (6.47) |  |  |
|  | Friesian | 16 | 3 (18.75) |  |  |

**Comment:** Please recheck the odds ratio as also suggested by one reviewer.

**Response:** the odds ration were checked and corrected

**Comment:** Finally, please evaluate the performance of RBT and i-ELISA considering culture/PCR the as gold standard.

**Response:** the comment was addressed as follows:

“The performance of RBT and i-ELISA indicated by the seroprevalence rate (2.7%) was low compared to the prevalence obtained by culture and ITS PCR (5.7%)”.

**Review Comments to the Author**

Please use the space provided to explain your answers to the questions above. You may also include additional comments for the author, including concerns about dual publication, research ethics, or publication ethics. (Please upload your review as an attachment if it exceeds 20,000 characters)

**Reviewer #1**: Dear Authors,

This is an interesting piece of work that will have an important implication on policy in Rwanda. I must congratulate the team for a well-conducted and written manuscript. However please double check odds ratio and its confidence interval which seems incomplete. All the best for your future.

“Thank You”

**Response:** Thank you so much. The odds ratio were checked and corrected.

**Reviewer #2:** Seroprevalence of brucellosis and characterisation of Brucella spp. from slaughtered cattle in Rwanda is well written paper about a very important zoonotic disease of human and animal health. Following are suggestions for improvement of paper before accepted for publication

**Response:** Thank you so much

**Comment:** Line 36-37. is test and slaughter policy applicable in Rwanda

**Response:** the comment was addressed as follows:

It is essential to urgently strengthen a coordinated national bovine brucellosis vaccination and initiate test-and-slaughter program which is not presently applicable in Rwanda.

**Comments:** Line 62-63. remove underline sign from species name of Brucella

**Response**: the underline sign was removed: PCR assays which differentiate *B. abortus* bv.1, 2, 4, *B. melitensis* bv.1, 2, 3, *B. ovis*, and *B. suis* bv.1 (AMOS PCR) in 24 hours from cultures

**Comment**: line 148, have you performed microscopy or biochemical testing for Brucella susceptable isolates

Response: we did not perform microscopy nor biochemical testing. All suspect cultures were screened with ITS PCR

**Comment:** Line 149, there is a need to add details of positive and negative controls used in genus specific PCRs

**Response:** the details for the positive and negative controls were indicated in line 153 as follows: **“**with the *B. abortus* RF 544 (Onderstepoort Biological Products, South Africa) as a positive control. For the negative control, we used ultrapure sterile water (Onderstepoort Biological Products, South Africa)”.

**Comment:** Line 179-180. what a was product length of B. abortus and melitensis in case of AMOS and Bru-ladder PCR assays-it will be a good addition, if you can add sequencing results too (if possible, otherwise you can try it next time)

**Response:** The expected product sizes in AMOS PCR assay are 498 bp and 731 bp for B. abortus and B. melitensis, respectively. The expected product sizes for B. abortus in Bruce-ladder PCR assay are 152 bp, 498 bp, 587 bp, 587 bp, and 794 bp; they are 152 bp, 498 bp, 587 bp, 794 bp and 1071 bp for B. melitensis.
