# Supplementary material for: Seroprevalence of brucellosis and molecular characterization of Brucella spp. from slaughtered cattle in Rwanda
Source: PLoS One. 2022 Nov 22;17(11):e0261595. doi: 10.1371/journal.pone.0261595 (PMC9681097; doi:10.1371/journal.pone.0261595)
Supplement: Attachment — Submitted filename: 2nd Response to reviewers -.docx [file pone.0261595.s003.docx]

**Response to reviewers**

**October 17^th^, 2022**

**Editor,**

**PLOS One**

**Re: revision of the manuscript [PONE-D-21-38427]-[EMID: 82b0bed3761197ba]**

Dear Editor

The authors would like to acknowledge your valuable input. We thank you for reviewing our manuscript entitled “**Seroprevalence of brucellosis and molecular characterization of *Brucella* spp. from slaughtered cattle in Rwanda**”. Your comments were very clear and useful to improve the quality of this manuscript. We have revised the manuscript considering all issues mentioned in your comments. Outlined below are the point-by-point responses.

**Comment:**

1. Please ignore my previous comment regarding the evaluation of RBT and i-ELISA considering culture/PCR as gold-standard as it was not performed properly and also it may not be possible to evaluate efficiently due small size of culture /PCR positive samples.

**Response:** the sentence was deleted

**Comment:**

2. Please estimate odds ratio appropriately. There should have one reference for every variables and usually the category with lowest prevalence is considered as reference. For example, Low throughput for abattoirs, eastern for provinces, etc.

**Response**: the univariable table was revised to remove the odds ratio. The variables with P ≤ 0.20 were selected for the multivariable analysis table.

Table 4. Results of the multivariable logistic regression analysis between animal characteristics and ITS PCR assay of *Brucella* spp. isolates from cultures of tissues of slaughtered cattle in Rwanda

| **Variables** | **Category** | **Odds Ratios** | **95% CI** | ***p*-Value** |
| --- | --- | --- | --- | --- |
| Breeds | Ankole ^a^ |  |  |  |
|  | Crossbreed | 5.7 | 0.73-44.06 | 0.097 |
|  | Friesian | 18.9 | 1.83-195.96 | 0.014 |

**Comment:**

3. Please replace "univariate" with "univariable" from Table 3 title.

**Response**: corrected

**Comment:**

4. Other than reference category, every category of a variable should have an odds ratio. What did you mean by "undetermined"?

**Response:**

**Comment:**

5. Please add before line 177: The brucellosis case status was defined based on the results of culture and ITS PCR tests in series.

**Response:** corrected

**Comment:**

6. Lines 177-180: Please add the following: The chi-square test was used to evaluate the univariable association between the explanatory variables and brucellosis case status. Any explanatory variable associated with brucellosis at a P≤0.20 was included in the multivariable logistic regression analysis to identify risk factors.

**Response**: sentences were added.

**Comment:**

7. Lines 222-223: The brucellosis case status was considered as prevalence based on bacterial growth and confirmation by ITS PCR. Please delete this sentence.

**Response:** the sentence was deleted

**Comment:**

8. Please delete lines 249-250

**Response:** corrected

**Comment:**

9. Please delete lines 334-346 as these results are not statistically significant.

**Response:** corrected

**Comment:**

10. The authors could add a discussion about the 70 samples which were culture positive but PCR negative

**Response:** All samples that presented growth on the CITA agar plate were subjected to ITS PCR for confirmation. The 70 samples were not culture-positive for *Brucella* and therefore can not be discussed.

**Comment:**

11. Please elaborate ITS and AMOS in the Tables 2 and 3 foot note

**Response:** corrected

**Comment:**

12. Line 348: Please replace "rate" with "prevalence"

**Response:** corrected
