# Supplementary material for: Seroprevalence of brucellosis and molecular characterization of Brucella spp. from slaughtered cattle in Rwanda
Source: PLoS One. 2022 Nov 22;17(11):e0261595. doi: 10.1371/journal.pone.0261595 (PMC9681097; doi:10.1371/journal.pone.0261595)
Supplement: Attachment — Submitted filename: 3rd Response to reviewers.docx [file pone.0261595.s004.docx]

**Response to reviewers**

**October 29^th^, 2022**

**Editor,**

**PLOS One**

**Re: revision of the manuscript [PONE-D-21-38427]-[EMID: 82b0bed3761197ba]**

Dear Editor

The authors would like to acknowledge your valuable input. We thank you for reviewing our manuscript entitled “**Seroprevalence of brucellosis and molecular characterization of *Brucella* spp. from slaughtered cattle in Rwanda**”. Your comments were very clear and useful to improve the quality of this manuscript. We have revised the manuscript considering all issues mentioned in your comments. Outlined below are the point-by-point responses.

**Comment:**

I still need clarification about one of my previous comment: The authors could add a discussion about the 70 samples which were culture positive but PCR negative

Response: All samples that presented growth on the CITA agar plate were subjected to ITS PCR for confirmation. The 70 samples were not culture-positive for Brucella and therefore cannot be discussed.

If this is the case then please clarify the figure 89 (29%) under the column 'culture'.

**Response:**

CITA medium and most other selective media focus on isolating *Brucella* but each medium still does not allow exclusive growth of *Brucella*. They reduce most contaminant bacteria but some others still grow. Thus other contaminants will grow on CITA and other selective media (Ledwaba et al., 2020). Considering the growth of contaminant bacteria that can even hide the *Brucella* growth, we screened each growth with ITS PCR for confirmation.

Figure 87 (29%) under column “culture” indicates bacterial growth including contaminants. Therefore, “culture” was changed to “bacterial growth” to avoid confusion.

**Comment:**

Please remove Table 4 as it is not based on the results of a multivariable model. A Multivariable model must contain at least two variables. As only one variable is associated with a p value <0.20 in the univariable screening there is no need to run the multivariable model. Please include odds ratio and their 95% Confidence interval in the Table 3.

**Response:**

The table was deleted.

Odds ratio and their 95% confidence interval were included in the univariable table.

| **Variables** | **Categories** | | **Tested** | | **ITS PCR assay on culture isolates** | | |  |  |
| --- | --- | --- | --- | --- | --- | --- | --- | --- | --- |
|  |  | |  | | **n^+^ (%)** | **Odds Ratio, 95% CI** | | ***p-*value** |  |
| Abattoir throughput | | Low (ref) | | 20 | | 1 (5.00) |  | |  |
|  |  | High | | 280 | | 16 (5.71) | 1.15 (0.14, 9.15) | | 0.894 |
| Provinces | | Eastern (ref) | | 70 | | 2 (2.86) |  | |  |
|  |  | Kigali city | | 30 | | 3 (10.00) | 3.78 (0.60, 23.88) | | 0.158 |
|  |  | Northern | | 50 | | 4 (8.00) | 2.96 (0.52, 16.81) | | 0.222 |
|  |  | Southern | | 80 | | 3 (3.75) | 1.32 (0.21, 8.16) | | 0.762 |
|  |  | Western | | 70 | | 5 (7.14) | 2.62 (0.49, 13.96) | | 0.261 |
| Age | | Young (ref) | | 31 | | 0 (0.00) |  | |  |
|  |  | Adults | | 269 | | 17 (6.32) | 7.80 (0, Inf.) | | 0.989 |
| Sex | | Males (ref) | | 13 | | 0 (0.00) |  | |  |
|  |  | Females | | 287 | | 17 (5.92) | 2.68 (0, Inf.) | | 0.989 |
| Breed | | Ankole (ref) | | 83 | | 1 (1.20) |  | |  |
|  |  | Cross | | 201 | | 13 (6.47) | 5.67 (0.73, 44.06) | | 0.0972 |
|  |  | Friesian | | 16 | | 3 (18.75) | 18.92 (1.83, 195.97) | | 0.0137 |

ref=reference level used in the analysis to compare infection; n+: number of positives; %: percentage, the 16S-23S ribosomal interspacer region (ITS), CI: confidence interval, inf.=infinity

Kind regards
